# Finding the Balance between Research and Monitoring: When Are Methods Good Enough to Understand Plastic Pollution?

**DOI:** 10.1021/acs.est.2c06018

**Published:** 2023-04-04

**Authors:** Amy L. Lusher, Sebastian Primpke

**Affiliations:** † Norwegian Institute for Water Research, Økernveien 94, 0579 Oslo, Norway; ‡ Department of Biological Sciences, University of Bergen, Thormølens Gate 53, 5008 Bergen, Norway; § Alfred-Wegener-Institute Helmholtz Centre for Polar and Marine Research, Biologische Anstalt Helgoland, Kurpromenade 201, 27498 Helgoland, Germany

**Keywords:** plastic litter, debris, environmental pollution, harmonization, microplastics

## Abstract

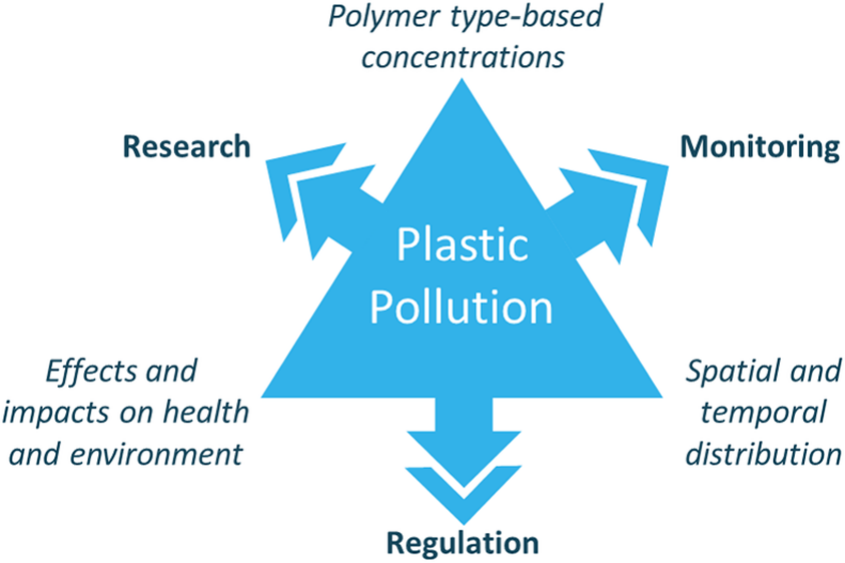

Plastic pollution is an international environmental problem. Desire to act is shared from the public to policymakers, yet motivation and approaches are diverging. Public attention is directed to reducing plastic consumption, cleaning local environments, and engaging in citizen science initiatives. Policymakers and regulators are working on prevention and mitigation measures, while international, regional, and national bodies are defining monitoring recommendations. Research activities are focused on validating approaches to address goals and comparing methods. Policy and regulation are eager to act on plastic pollution, often asking questions researchers cannot answer with available methods. The purpose of monitoring will define which method is implemented. A clear and open dialogue between all actors is essential to facilitate communication on what is feasible with current methods, further research, and development needs. For example, some methods can already be used for international monitoring, yet limitations including target plastic types and sizes, sampling strategy, available infrastructure and analytical capacity, and harmonization of generated data remain. Time and resources to advance scientific understanding must be balanced against the need to answer pressing policy issues.

## Abstract 2

Interdisciplinary action on plastic pollution requires clear dialogue and balanced understanding of aims, available methods, and expected outputs.

Plastics are increasingly reported in environmental samples across the globe. This ubiquitous and heterogeneous environmental contaminant is receiving attention from researchers, citizens, and policymakers. Monitoring plastic pollution is positioned high on agendas of international governing bodies, with the realization of a new legally binding global instrument on plastic pollution during the Fifth Session of the United Nations Environment Assembly (UNEA 5.2). This is mirrored by calls from regional and national agencies to understand the extent of this pollutant in their local environments. Much attention is directed toward the perceived harm plastic pollution could cause to the environment, including animals and humans.^1,2^ It is imperative that any potential risk is assessed appropriately and that mitigation measures can be monitored accordingly.

Monitoring is necessary to address questions about the presence and abundance of plastics in the environment. As monitoring is the repeated measurement of variables to detect a change,^3^ it requires significant data gathering, analysis, and archiving. This cannot be achieved until appropriate approaches are chosen to address well-defined goals which should be subject to regular review.^4^ Frameworks and instruments to conduct environmental assessments must be ratified with a clear purpose before monitoring can be initiated. For example, UNEA supports many activities concerning marine litter with an emphasis on monitoring through Resolution 4.6.d. EU Member States are required to report on the quality status of their marine and freshwater bodies under the Marine Strategy Framework Directive (MSFD) and Water Framework Directive (WFD). Work is ongoing within EU Member States and Regional Sea Conventions (i.e OSPAR, HELCOM, etc.) to formalize plastic monitoring frameworks and instruments—including stringent environmental assessment practices—according to national and regional circumstances.^5^ Individual countries will be required to identify mechanisms through which monitoring will be carried out.

Environmental monitoring is currently facing a global challenge to generate reliable and comparable data on plastic pollution. This limitation is driven by how the need for monitoring is interpreted. Approaches to monitoring and choice of matrix or indicators, as well as reporting criteria, are currently being interpreted in different ways by key actors (national and international expert working groups, scientists, and policymakers). This has led to diverse approaches being adopted around the world. Of those instruments in place to begin monitoring (i.e., Descriptor 10 - EU MSFD), methods used to analyze plastics (especially microplastics) tend to vary in terms of sampling, sample extraction, identification, and data reporting.^6−11^ Challenges posed by choice of methodological approach, as well as access to infrastructure to perform analysis, further complicate the matter. Subsequently, data on plastic presence in the environment vary in quality, resolution, and focus.^12^ This compromises comparative assessments and limits confidence related to impacts of plastic pollution.^13−15^ It is essential to undertake major actions for the evaluation and optimization of plastic pollution monitoring and assessment to reach substantial improvements in environmental sustainability and socio-economic development. Importantly, method limitations should be addressed including (1) what can be achieved now and (2) where should time and research be dedicated to improve methodologies.

The lack of a harmonized, or common voice, with regard to choosing environmental matrices or analytical approaches is slowing ratification of monitoring frameworks. The demand for data so that policy can act comes with reservations. In some instances, methods are not yet considered suitable for the questions being asked. Another problem emerges when questions are unspecific or interpretable in the context of research and monitoring. They may require further methodological advancements. Such advances are money dependent, and high-end methods require investment into approaches that are still in the development phase and not accessible to all. Research purposes, aims, and objectives must be well defined when identifying methods. It is unsuitable, for example, that risk assessment for human health relies mainly on data from larger particles. Another example of unsuitable data use would be calculations of mass balances based solely on particle number and size as they rely on assumptions related to a mass estimation.^9,16^

Robust and harmonized approaches are required to reinforce existing initiatives and improve coordination. Efforts extending from national to global levels will facilitate efficient risk assessment and environmental policy implementation. Through this perspective, we address the issue of finding a balance between research and development, regulation, and monitoring within the field of plastic pollution. Considering that each of these elements comes with individual targets regarding data needs, we focus on bringing research into monitoring by asking the following: (1) Why and what should we monitor? (2) Are there methods available to provide environmental baseline levels, and how should data be reported? (3) When should novel and developing techniques be implemented? (4) What frameworks are available to ensure a comparative approach is adopted between national and international organizations? (5) How do we balance and align scientific advancement with requirements from policymakers and regulators to obtain a rapid response? Our aim is to discuss complexities of answering calls from governing organizations to identify environmental contamination from plastic pollution.

## Why and What Should We Monitor?

Monitoring is usually performed to understand impacts or effects of an environmental variable, for example, a specific pollutant or temperature changes. Defining what to monitor and the extent of change one would like to detect determines what is sampled and the amount of sampling needed.^17^ For plastic pollution, the purpose is to understand levels in the environment or to determine values before (baseline) and after intervention, such as those related to mitigation or remediation measures. Whichever the purpose, monitoring should be built around the principles of reproducibility, replicability, and repeatability.^18^

Monitoring requirements are usually initiated by the needs of an entity. On a national level, this is usually environmental agencies acting in response to national or international regulations. Reaction to a monitoring demand must be fast. Often, there is little time to develop new methods. Researchers therefore must define which methods are suitable and any limitations they may have. For example, there are robust methods for identifying plastics on beaches^19−21^ which have been adopted for monitoring under OSPAR and more recently AMAP.^22−25^ Still, for microplastics, such approaches and their limitations have only been rigorously assessed in few cases. Once an approach to monitoring is identified, it must be linked to the question or purpose. For example, it makes no sense to calculate polymer masses if particle numbers are needed and vice versa. Likewise, if monitoring is to identify risk of human exposure to plastics in seafood, the matrix sampled cannot be inedible tissues. Further, when smaller microplastics (<100 μm) are the target of an assessment (for example, drinking water), the methodological approach must facilitate accurate identification.

There are many options when identifying which environmental matrix to sample for plastic pollution. The choice should align with the needs of an entity yet be informed by the state of the research field (i.e., method availability, ability to detect a change). As an example, plastics which are found in marine environmental matrices have high spatial and temporal variability. On the sea surface, plastics may accumulate in oceanographic features (e.g., ref (26)), but they are quickly transported from source or release locations,^27^ making it difficult to ascertain their origin. It is easy to gather data on large plastic items washed up on beaches; however, the interpretation is complex. The dynamic nature of plastic fluxes on beaches is further complicated by increased cleaning efforts in recent years—impeding temporal data assessment.^4^ Biota appear to be useful indicators of plastic interactions; however, much of the sampling is destructive. Sampling stranded or dead individuals also comes with bias, so an ethical approach is required. It is therefore important that studies take a cross-environmental, multi-indicator focus approach to ascertain the most complete picture of plastic pollution.^28^

Plastic size adds complexity to what can be monitored and has several constraints. First, the design of sampling devices and methods must be different for small-sized particles as they cannot be accurately collected by, for example, manta trawl nets. Similarly, sample processing is more demanding and time consuming as specialized filters and handling are required. Not surprisingly, the ability of researchers to detect smaller particles is correlated to methods applied, laboratories’ experiences, and ability to work with small-sized particles (e.g., ref (29)). Size also influences the impact of the derived data. Taking macroplastics as an example, one can detect a composition change in sources of plastics identified on beaches. Ryan et al.^4^ suggested monitoring should estimate flows of materials rather than standing stocks because sufficient understanding of turnover rates in environmental compartments is lacking. They also suggest that monitoring plastics smaller than 1 mm is not recommended as methods are not yet available.^4^ Further, impacts of macroplastics—mostly related to physical impacts toward an individual or an environment (entanglement, ingestion, smothering etc.) or economic impacts (littering and clean ups)—are far easier to infer sources, such as fishing debris entanglement or user-derived littering. It is easy to determine when an item is plastic, and in many cases, one can point toward a source category (fishing, shipping, recreational activities, littering, etc.). Available methods do not require sophisticated analytical approaches. However, when investigating risks of smaller-sized particles,^2,13,14^ an imbalance remains between methods applied to environmental samples compared to those used in ecotoxicological approaches. Polymers can be detected, but source apportionment cannot generally be achieved. This complicates environmental risk assessments required to outline risks associated with exposure and provide the justification for mitigation and remediation actions.

For research to react to policy requirements, we must assess if approaches applied will answer questions being posed in the current place and time. Taking a step back and observing the rapid pace and change in our approach to understanding plastic pollution puts this interesting conundrum into context. Method development for addressing plastic pollution began with a focus on the ocean. The marine environment has long been identified as the ultimate end point for plastic pollution, transported from land to sea. Since receiving much attention over the past decades, some advancement has been made toward establishing harmonized methodologies. Methods for macroplastic observations (seafloor/sea surface) and ingestion of plastics by seabirds are now commonplace (e.g., refs (5, 30, 31)). However, freshwater systems and the atmosphere are sources and receivers of plastic pollution. They are also important compartments connecting terrestrial sources of plastics to the ocean. Attention must be addressed to these systems and associated transport pathways to effectively tackle global plastic pollution. Many methods used in the marine system are transferable, with some modifications, to freshwater and terrestrial systems.

## Are There Methods and Data Reporting Tools Already Available to Provide Baseline Levels in the Environment?

The requirement to begin monitoring can be hindered by researchers’ drives and curiosities to push boundaries of methodological approaches. Monitoring should begin now. Changes in methods should not prevent this. Instead, validation and feasibility studies should be conducted so that comparative approaches can be adopted. Similar methods for large plastics and marine litter are already being implemented, reflecting their early inclusion in monitoring recommendations (e.g., refs (5, 17)). Methods for microplastic analysis reached a certain baseline level of suitable sampling, extraction, and identification tools in recent years (e.g., refs (6, 8−10)). Still, many techniques are targeted by method optimization approaches and improvement in the speed of analysis. Major limitations of all these methods are costs of instrumentation and personal, as well as expenditure of time per sample.

For example, sampling procedures for water bodies narrow down to mainly net sampling, pump filtration, or filtration cascades. In these cases, choice of sampling device can already be determined by the monitoring or research question. As an example, net sampling is sufficient to monitor larger items (>300 μm) typically ingested by birds and larger marine animals, while it would not be sufficient for determining exposure risks for humans. In this case, a filter cascade or filtration pump will be more suitable. Differences in results obtained by various systems are currently under investigation by many working groups. Second, sample preparation (clean up and microplastic extraction) can be narrowed down to a few high-performance approaches to remove organic material such as hydrogen peroxide, Fenton’s reagent, potassium hydroxide, or enzymatic degradation (see ref (8) for detailed assessment). Similarly, various high-density salt solutions (e.g., ZnCl_2_, NaBr) can be used to isolate microplastics from sediments, with priority given to a salt solution which can be recycled for environmental and economic reasons.

Identification of microplastics and data reporting follow as a last step of the pipeline. Here, various methods are available using optical investigation via stereomicroscopy or a dye-staining supported analysis.^9^ Characterization of associated chemicals is also of emerging concern for risk assessment.^32^ In general, two types of analytical principles are currently in use which allow for either individual particle-based (spectroscopy) or polymer mass-related (thermoanalytical) data to be derived. These techniques can be combined with optical identification techniques for preselected particles or combined for spectroscopic applications. While the details and sensitivity of the individual methods are being compared, the general measurement principles of the instruments are mostly harmonized already. For example, FTIR analysis can be performed for data sets from four different instruments by the same software tool with relative ease,^33^ such that sufficient data can be obtained regarding shape, size, polymeric structure, and color. Currently, FTIR is still the most widely applied technique.^9^ However, a barrier exists when the set up (and activation) of new instrumentation is hindered by the need for training and instrument optimization for working with plastics. Nevertheless, data quality suitable for monitoring can already be achieved by following available guidelines.^6,34^

Compared to the discussion about the available analytical methods and tools, the urgent need for suitable data reporting styles and platforms is prominent in expert committees working on monitoring guidelines and standardization efforts. While there is a high demand for such possibilities,^35^ the current solutions are not completely harmonized with monitoring needs. In addition, reporting and storage of abundance (number of particles per *xyz*) data in combination with metadata such as color, shape, and size are favored. Broader data reporting may overcome most current concerns of scientists regarding characterization methods. Similarly, techniques are available to collect detailed visual images and chemical fingerprints of particles. Therefore, databases for reporting should also allow inclusion of nonidentified particles to enable researchers to reassess data sets and perform data analyses with improved methods in the future. This approach combines the demands for starting monitoring early with the ability to still improve methodologies used for identification, thus allowing baseline assessments for microplastics to begin.

## When Should Novel and Developing Techniques Be Implemented?

Monitoring requires robust and tested methods that can produce comparable results irrespective of their implementation. This requires intercalibration and validation between monitoring organizations and must be addressed internationally. When a suitable method is identified for monitoring, this should not see the end of scientific advances in methodological approaches. Researchers working in fields like analytical chemistry are driven by their curiosity to push boundaries of their science, rather than the need to monitor a particular pollutant. Other fields of research are interested in the very details of the problem evolving from microplastics investigations having different data demands compared to monitoring. Here, it is most important to consider that currently emerging methods—still in the design or test phase—cannot be implemented until they are rigorously tested and validated against already in-use methods. Otherwise, compatibility between data collected with one method versus another may be affected and compromise assimilation of long-term or temporal data sets.

Most monitoring programs allow for method improvements, providing they have been through rigorous tests. Compared to advancements in fields of analytical chemistry, it is of higher importance that plastic pollution monitoring programs are designed to be statistically robust. Analysis is not targeting a known compound with specific chemical properties (such as persistent, bioaccumulative, and toxic chemicals, PBTs); instead, plastics are a heterogeneous mix of sizes, shapes, polymers, and chemical compositions. Numbers of samples and volume sampled are therefore of significance. A power analysis can be used to determine the number of sites, as well as intensity and frequency of sampling at each site.^36,37^ Similarly, strategies must be in place to allow an assessment into the feasibility of new methods and validation against comparative approaches.

One example is the use of manta nets to collect plastics from surface waters. This methodological approach has long been used within plastic research, and it is a recommended tool for monitoring (e.g., refs (17, 22, 38)). A manta net can obtain replicable data for particles >300 μm, although it is limited by use in coastal areas and highly weather dependent.^28^ Manta nets can be used if limitations are clearly acknowledged. For example, underrepresentation of particles <300 μm has been highlighted as a problem.^39^ Improvements or modifications of this sampling approach would be the inclusion of smaller mesh sizes or the use of filter cascades to allow comparable data at 300 μm and inclusion of smaller particles.

## Frameworks to Ensure a Comparative Approach Is Adopted by National and International Organizations

Introducing best available practices that are harmonized and validated has become critical for the coherence of monitoring within framework policies, such as EU MSFD, WFD, and other international agreements. Thus, harmonizing approaches is an important step for coordinating future activities under marine, freshwater, and terrestrial directives. A coordinated approach which brings key actors together will be vital.

Harmonization in the field of plastic pollution is considered as the development of a cluster of monitoring procedures—including sampling strategy, sample collection, handling and storage, sample preparation, analysis, quality assurance and control criteria, and data management protocols—which provide cross-comparable data. Such methods should be assessed for their feasibility through validation approaches. Obstacles for optimized and harmonized monitoring include biological, environmental, methodological, logistical, analytical, and ethical constraints.^40^ Not all methods are suitable for different environmental matrices or plastic sizes nor are they accessible for all researchers around the world.

Accessibility underpins any globally harmonized approach to understanding plastic pollution. Methods must be suitable and available for any participant or organization engaged in monitoring. High-end instrumentation is too costly for the global south. Training and capacity building (e.g., the Horizon Europe Twinning program *GREENLand – Microplastic-free environment*, project-greenland.com) should be made available on a broader scale if international approaches demand higher resolutions in data.

Any approach to monitoring should undertake an assessment of available methods, their use across different matrices, their comparability to one another, and the accessibility for regional use. It is fundamental to validate methods before implementation into monitoring frameworks to ensure harmonization. Carefully selecting and validating methods using a feasibility assessment, which is heavily rooted in quality assurance and quality control (QA/QC), will identify any uncertainties in methodological approach, cost effectiveness, and comparability between methods. Harmonized methodological approaches will enable regulatory compliance by different private and public actors and the ability to assess the effectiveness of environmental protection policies.

## Balancing and Aligning Scientific Advancement with Requirements from Policymakers and Regulators for a Rapid Response

A thorough assessment of methods available to monitor plastics and microplastics in different environmental matrices is urgently required. A systematic approach to conducting an assessment on the forever expanding scientific and gray literature is needed. Some methods are ready to be implemented globally and have already shown they are effective for long-term monitoring. These methods include assessments of beach litter (e.g., ref (4)), floating mesoplastics (e.g., ref (37)), and plastics ingested by seabirds (e.g., ref (30)). However, other methods such as those used for assessing microplastics in the atmosphere, are still undergoing research and development and not yet ready for monitoring on a broader scale.^40,41^

While research aims to achieve a more detailed understanding of plastic spatial and temporal distribution, monitoring aims to generate feedback on the status of an environmental compartment. In contrast, risk assessment is mainly looking for target sizes and polymer types which have been linked to effects on environmental and human health. Each aim has different demands for spatial scale and data reporting. While data from research can often be reduced to data sets applicable to risk assessment and monitoring,^16^ this may be hampered contrariwise if similar data were not collected. To find a balance, minimum aspects should be defined by research, risk assessment, and modeling on various parameters available.

Minimum reporting requirements begin with definitions of particle types, colors, and size classes (e.g., refs (8, 42, 43)). Accordingly, the AMAP guidelines for litter and plastic monitoring tried to implement a common basis for the Arctic by defining reported data into three size classes, color coding, and polymer type categories.^22^ Rather than using fixed values for small-sized particles, reporting demands definition of the lower size limit measurable in the data set. Such an approach covers various aspects of harmonization by defining a common ground for data reporting, allowing data evaluation (e.g., risk assessment and research for suitability of their tasks) and scientific advancement (e.g., toward smaller-sized particles). By reporting in a common database, entries may be linked to data repositories containing the full resolution data set of determined polymers, individual particle sizes, broader range of color codes, and other details with relative ease. Using a common basis for data generation, scientific advancement can easily be woven into tools providing a rapid response to policy makers and regulators. Further, by regularly updating monitoring guidelines and standards, methodology changes can be implemented on a regular basis into these and respective data reporting tools.

## Conclusion

**Figure 1:**
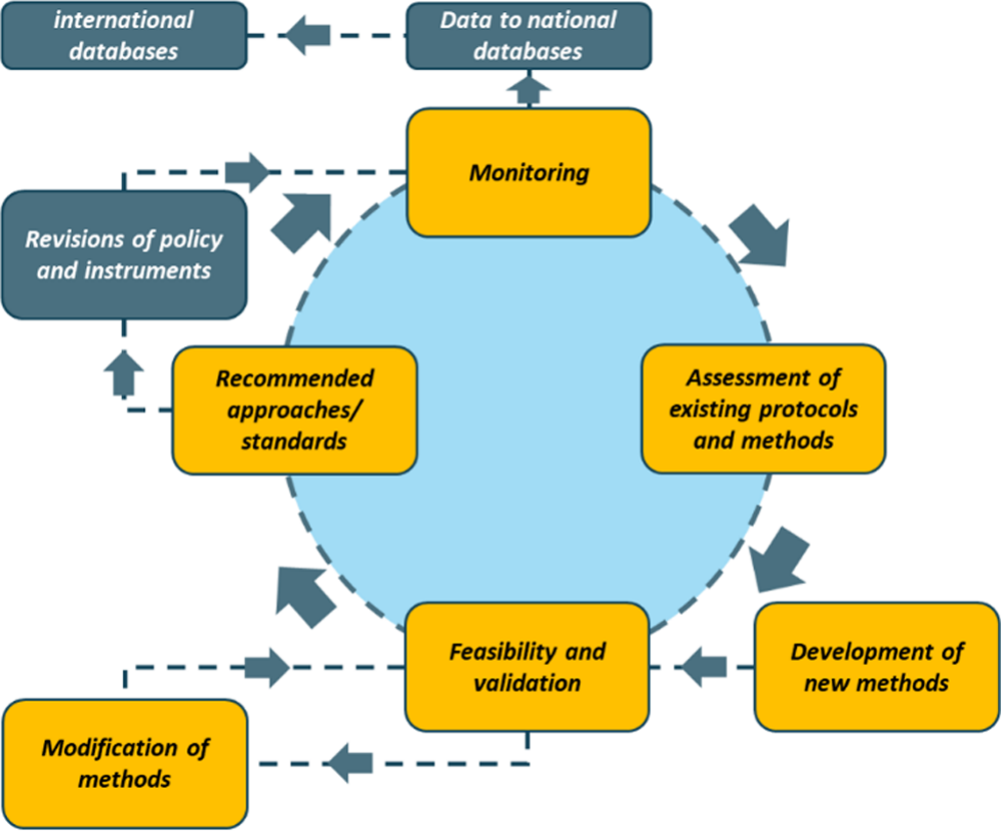
Schematic of a continuous loop assessment for the development and implementation of methods within the context of environmental plastic pollution monitoring. Information ascertained from assessing existing methods and new developments in methods can be tested for feasibility, and validated before recommending approaches. When data are generated, it can be sent to national and international databases.

A pragmatic, balanced, and open approach is needed when addressing requirements for monitoring while still facilitating scientific advancement. When policy frameworks require monitoring, chosen approaches should utilize existing protocols that have been tested for feasibility and validated between institutes and countries. When methods are developed, they should be assessed similarly, and modifications should be validated before such protocols are recommended for revisions of policy. This should be a continuous loop encouraging the development of new methods and testing method feasibility and validity, before recommending approaches for monitoring (Figure 1).

## Abbreviations

AMAP: Arctic Monitoring and Assessment Programme
MSFD: Marine Strategy Framework Directive
UNEA: United National Environment Assembly
WFD: Water Framework Directive
